# Supplementary material for: The eEF2 kinase coordinates the DNA damage response to cisplatin by supporting p53 activation
Source: Cell Death Dis. 2024 Jul 13;15(7):501. doi: 10.1038/s41419-024-06891-4 (PMC11246425; doi:10.1038/s41419-024-06891-4)
Supplement: Supplementary file 1 — Supplementary information [file 41419_2024_6891_MOESM1_ESM.docx]

**supplemental Figure legendS**

**Supplementary Figure 1. Related to Figure 1. Validation of eEF2K knockout and knockdown, and levels of eEF2 phosphorylation under cisplatin treatment**

**A.** eEF2K expression levels in *Eef2k*^+/+^ and *Eef2k*^-/-^ MEFs, as measured with immunoblot analysis using the indicated antibodies. **B.** eEF2K expression levels in HEK293 cells stably expressing individual shRNAs targeting eEF2K (sh-eEF2K1 and sh-eEF2K2) or scrambled control (sh-scr), as measured with immunoblot analysis using the indicated antibodies. **C.** Level of eEF2 phosphorylation (p-eEF2) in HEK293 cells treated with CisPt (5 μM) for the indicated times, as measured with immunoblot analysis using the indicated antibodies.

**Supplementary Figure 2. Related to Figure 3. Cell cycle analysis and ERCC1 expression of eEF2K-deficient cells versus control cells under cisplatin treatment**

**A.** Cell cycle analysis of *Eef2k*^+/+^ and *Eef2k*^-/-^ MEFs treated with CisPt (5 μM) for the indicated times, as measured by propidium iodide (PI) staining. **B.** ERCC1 expression levels in MEFs transfected with siRNA targeting eEF2K (si-Eef2k) or scrambled control (scr) treated with CisPt (5 μM) for the indicated times, as measured with immunoblot analysis using the indicated antibodies. **C.** ERCC1 expression levels in HEK293 cells stably expressing individual shRNAs targeting eEF2K (sh-eEF2K1 and sh-eEF2K2) or scrambled control (sh-scr) treated with CisPt (5 μM) for the indicated times, as measured with immunoblot analysis.

**Supplementary Figure 3. Related to Figure 4. p53 mRNA levels of eEF2K-deficient and control MEFs under cisplatin treatment**

**A.** Normalized p53 mRNA levels in *Eef2k*^+/+^ and *Eef2k*^-/-^ MEFs treated with 50 μM CisPt for the indicated times, as measured with qRT-PCR. **B.** Level of total p53 protein in *Eef2k*^+/+^ cells transfected with siRNA targeting p53 (si-p53-1 and si-p53-2) or scrambled control (scr), as measured with immunoblot analysis using the indicated antibodies. Data are expressed as mean ± SD; **P* < 0.05, ****P* < 0.005.
