# Supplementary figures and images for: The eEF2 kinase coordinates the DNA damage response to cisplatin by supporting p53 activation
Source: Cell Death Dis. 2024 Jul 13;15(7):501. doi: 10.1038/s41419-024-06891-4 (PMC11246425; doi:10.1038/s41419-024-06891-4)

A.

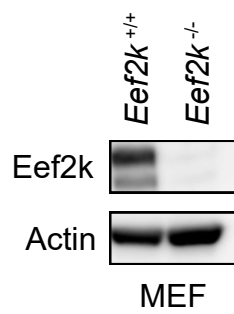

B.

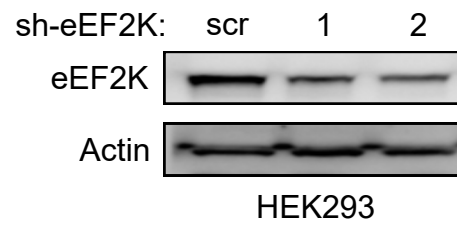

C.

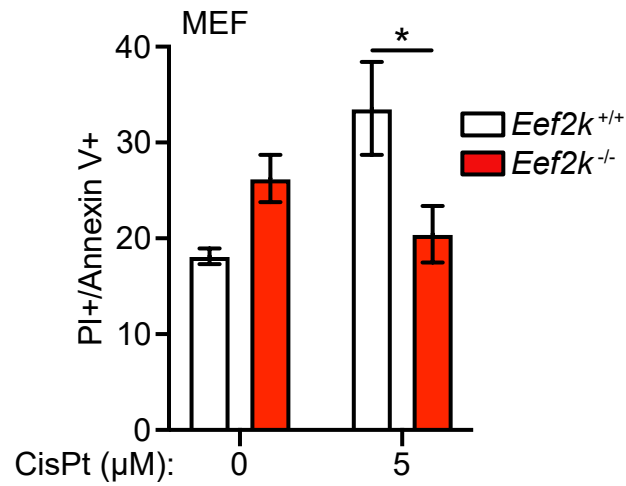

D.

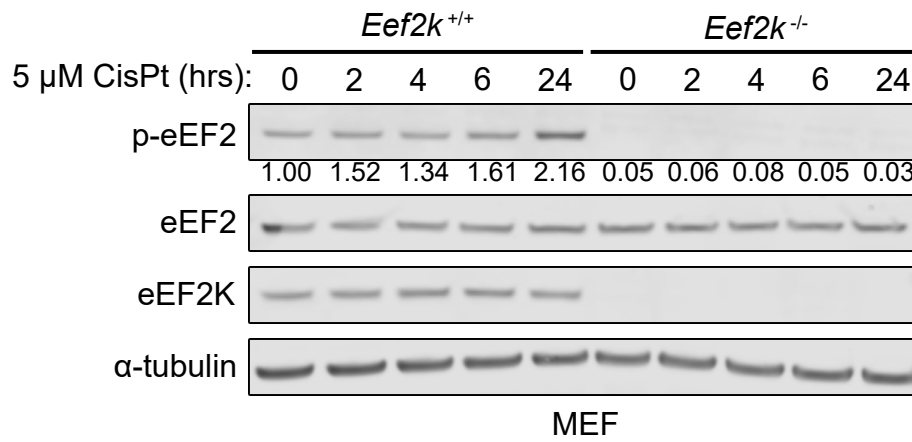

E.

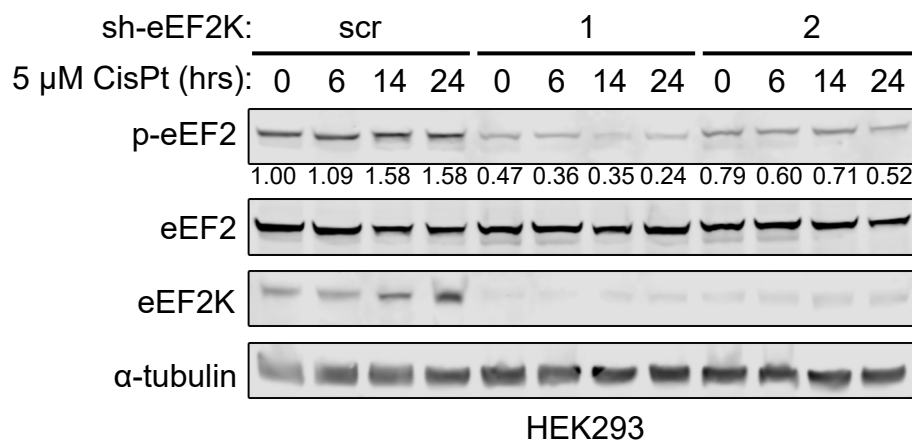

Supplement: Supplementary file 2 — Supplementary Figure 1 [file 41419_2024_6891_MOESM2_ESM.pdf]

A.

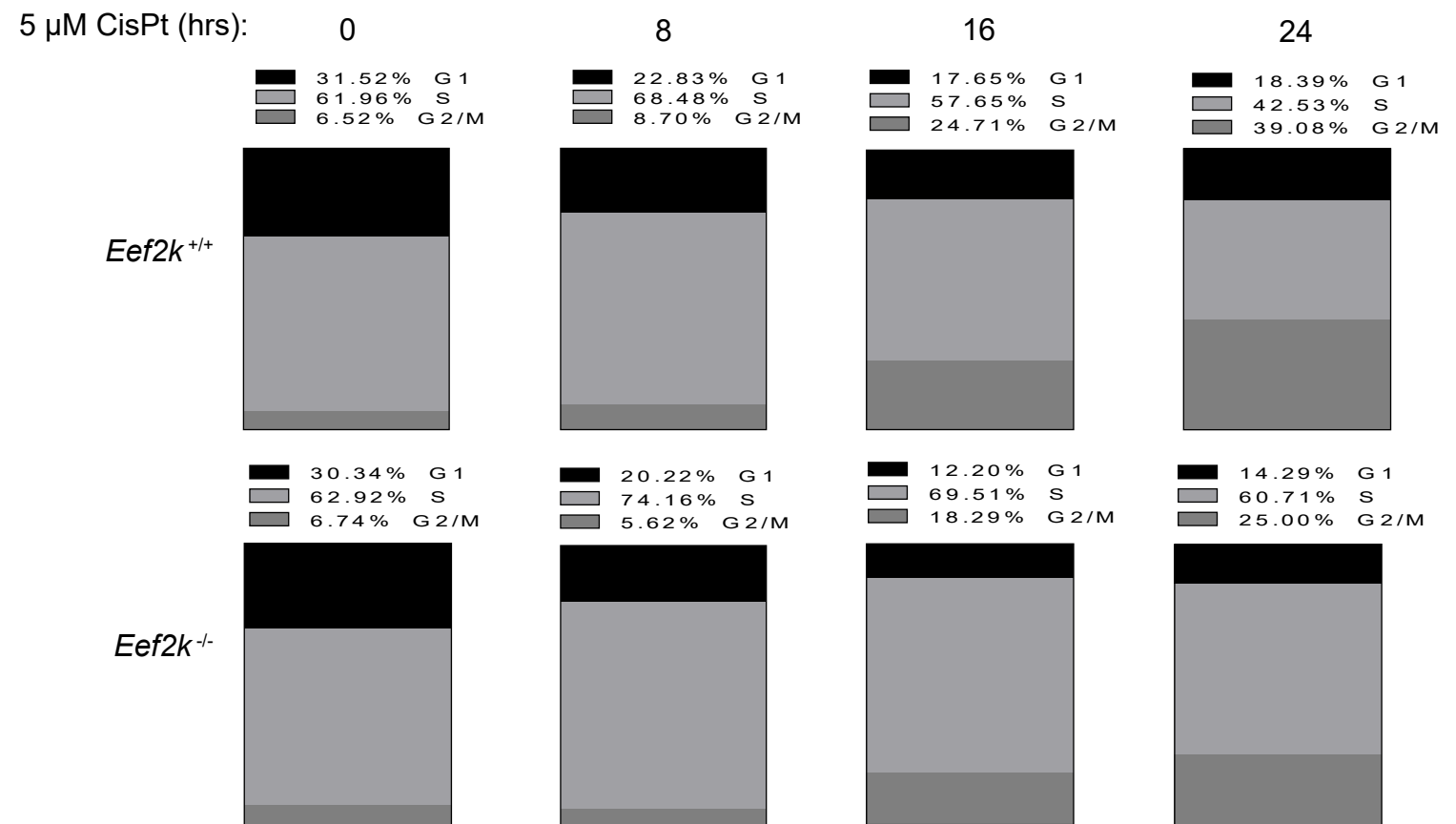

B.

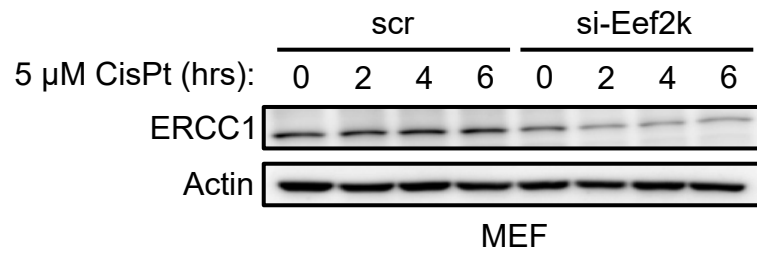

C.

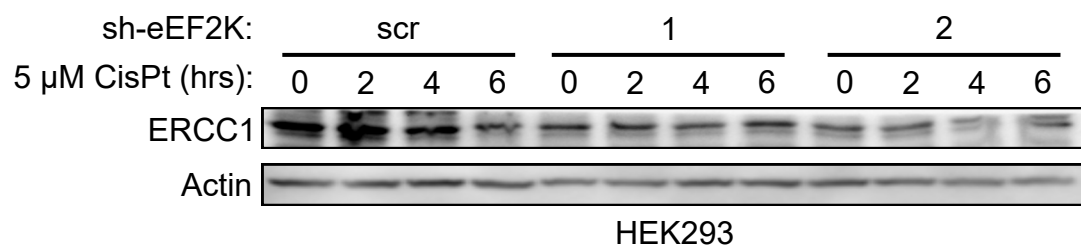

D.

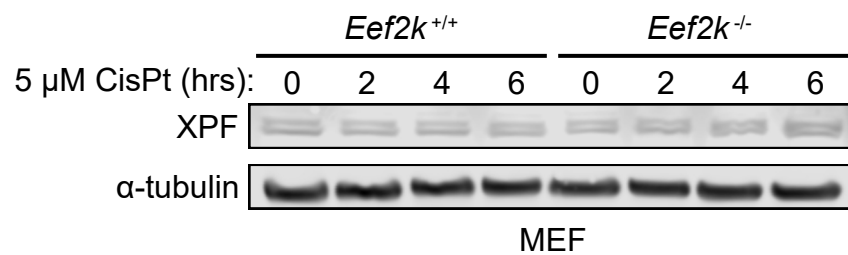

Supplement: Supplementary file 3 — Supplementary Figure 2 [file 41419_2024_6891_MOESM3_ESM.pdf]

A.

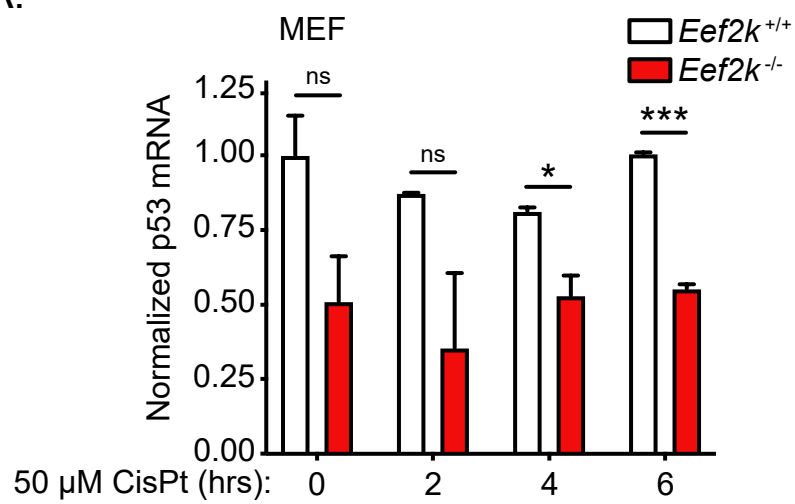

B.

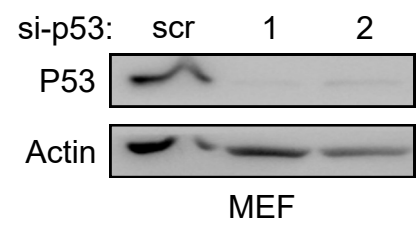

C.

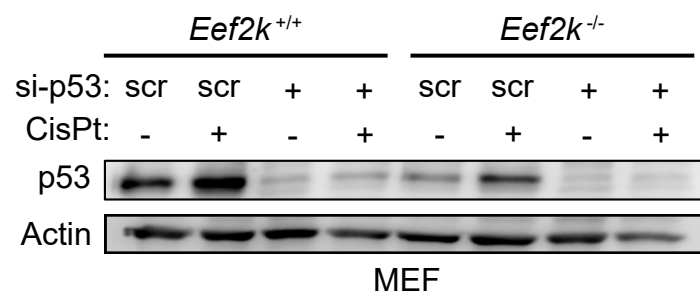

Supplement: Supplementary file 4 — Supplementary Figure 3 [file 41419_2024_6891_MOESM4_ESM.pdf]
